# 4,4′-(Propane-1,3-di­yl)dibenzoic acid

**DOI:** 10.1107/S1600536809033005

**Published:** 2009-08-26

**Authors:** Jia Hua, Lu Gao

**Affiliations:** a State Key Laboratory of Inorganic Synthesis and Preparative Chemistry, College of Chemistry, Jilin University, Changchun 130012, People’s Republic of China

## Abstract

The complete molecule of the title compound, C_17_H_16_O_4_,  is generated by crystallographic twofold symmetry, with the central C atom lying on the rotation axis and a dihedral angle between the benzene rings of 81.9 (2)°. In the crystal, mol­ecules are linked by O—H⋯O hydrogen bonding between carboxyl groups, forming one-dimensional supra­molecular chains.

## Related literature

For general background, see: Bradshaw *et al.* (2005 ▶); Eddaoudi *et al.* (2001 ▶); Heo *et al.* (2007 ▶); Kesanli & Lin (2003 ▶). For related structures, see: Dai *et al.* (2005 ▶); Li *et al.* (2007 ▶); Ma *et al.* (2006 ▶). For the synthesis, see: Cram & Steinberg (1951 ▶).
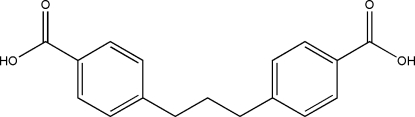


## Experimental

### 

#### Crystal data

C_17_H_16_O_4_
*M* *_r_* = 284.30
Monoclinic, 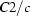

*a* = 14.569 (3) Å
*b* = 4.7337 (6) Å
*c* = 21.463 (3) Å
β = 102.722 (10)°
*V* = 1443.8 (4) Å^3^
*Z* = 4
Mo *K*α radiation
μ = 0.09 mm^−1^
*T* = 298 K
0.48 × 0.20 × 0.16 mm

#### Data collection

Bruker SMART CCD area-detector diffractometer
Absorption correction: multi-scan (*SADABS*; Sheldrick, 2004 ▶) *T*_min_ = 0.947, *T*_max_ = 0.989
3830 measured reflections
1276 independent reflections
688 reflections with *I* > 2σ(*I*)
*R*_int_ = 0.059

#### Refinement

*R*[*F* ^2^ > 2σ(*F* ^2^)] = 0.040
*wR*(*F* ^2^) = 0.115
*S* = 0.94
1276 reflections
96 parameters
H-atom parameters constrained
Δρ_max_ = 0.11 e Å^−3^
Δρ_min_ = −0.17 e Å^−3^

### 

Data collection: *SMART* (Bruker, 2001 ▶); cell refinement: *SAINT-Plus* (Bruker, 2003 ▶); data reduction: *SAINT-Plus*; program(s) used to solve structure: *SHELXS97* (Sheldrick, 2008 ▶); program(s) used to refine structure: *SHELXL97* (Sheldrick, 2008); molecular graphics: *ORTEP-3 for Windows* (Farrugia, 1997 ▶); software used to prepare material for publication: *WinGX* (Farrugia, 1999 ▶).

## Supplementary Material

Crystal structure: contains datablocks global, I. DOI: 10.1107/S1600536809033005/xu2559sup1.cif

Structure factors: contains datablocks I. DOI: 10.1107/S1600536809033005/xu2559Isup2.hkl

Additional supplementary materials:  crystallographic information; 3D view; checkCIF report

## Figures and Tables

**Table 1:** Hydrogen-bond geometry (Å, °)

*D*—H⋯*A*	*D*—H	H⋯*A*	*D*⋯*A*	*D*—H⋯*A*
O2—H2⋯O1^i^	0.82	1.84	2.642 (2)	168

Symmetry code: (i) 
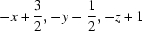
.

**Fig. 1.**
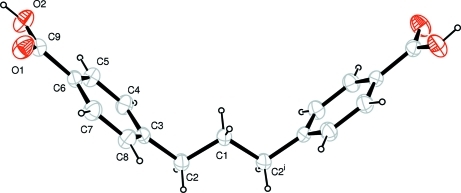
The molecular structure of the title compound with displacement ellipsoids at 50% probability level [symmetry code: (i) 1 - x, y, -z + 3/2].

**Fig. 2.**
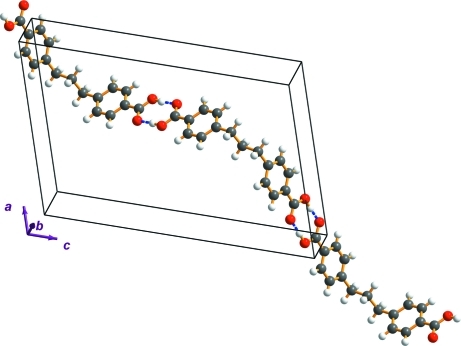
A diagram showing the one-dimensional supra-molecular chain formed by intermolecular O—H···O hydrogen bonding (dashed lines).

## supplementary crystallographic information

### Comment

In the past decades the design and synthesis of metal-organic frameworks have received extensive attention in the field of supra-molecular chemistry and crystal engineering (Bradshaw *et al.*, 2005; Eddaoudi *et al.*, 2001; Heo *et al.*, 2007; Kesanli *et al.*, 2003). As part of our investigation on the metal-organic frameworks (Dai *et al.*, 2005; Li *et al.*, 2007; Ma *et al.*, 2006), we report here the crystal structure of the title compound.

In the crystal structure, the title molecule has site symmetry 2, the C1 atom is located on a twofold rotation axis. The two symmetry-related benzene rings are twisted with respect to each other with a dihedral angle of 81.9 (2)° (Fig. 1). The carboxylic acid groups of neighboring molecules form strong intermolecular O—H···O hydrogen bonds (Table 1), linking the molecules into the one-dimensional supra-molecular chains (Fig. 2).

### Experimental

4,4'-(Propane-1,3-diyl)dibenzoic acid was synthesized according to literature methods (Cram & Steinberg, 1951), and other reagents were commercially obtained without further purification. In a typical synthesis procedure for the title compound, the reactants Zn(NO_3_)_2_.6H_2_O (0.149 g, 0.5 mmol), 4,4'-(propane-1,3-diyl)dibenzoic acid (0.142 g, 0.5 mmol), HCl (38%, 0.30 ml) and triethylamine (0.35 ml) were mixed in water (5 ml). The mixture was placed in a 15 ml Teflon-lined stainless steel autoclave with a filling capacity of 37.7% and heated under autogenous pressure for 5 d at 453 K. After slow cooling to room temperature, the block-shaped colourless crystals suitable for X-ray diffraction were obtained.

### Refinement

H atoms were positioned geometrically with C—H = 0.93 (aromatic), 0.97 (methylene) and O—H = 0.82 Å, and allowed to ride on their parent atoms with *U*_iso_(H) = 0.08 Å^2^.

### Crystal data

**Table:**

C_17_H_16_O_4_	*F*(000) = 600
*M**_r_* = 284.30	*D*_x_ = 1.308 Mg m^−^^3^
Monoclinic, *C*2/*c*	Mo *K*α radiation, λ = 0.71073 Å
Hall symbol: -C 2yc	Cell parameters from 750 reflections
*a* = 14.569 (3) Å	θ = 2.9–24.2°
*b* = 4.7337 (6) Å	µ = 0.09 mm^−^^1^
*c* = 21.463 (3) Å	*T* = 298 K
β = 102.722 (10)°	Block, colourless
*V* = 1443.8 (4) Å^3^	0.48 × 0.20 × 0.16 mm
*Z* = 4	

### Data collection

**Table:**

Bruker SMART CCD area-detector diffractometer	1276 independent reflections
Radiation source: fine-focus sealed tube	688 reflections with *I* > 2σ(*I*)
graphite	*R*_int_ = 0.059
φ and ω scans	θ_max_ = 25.0°, θ_min_ = 2.0°
Absorption correction: multi-scan (*SADABS*; Sheldrick, 2004)	*h* = −11→17
*T*_min_ = 0.947, *T*_max_ = 0.989	*k* = −5→5
3830 measured reflections	*l* = −25→25

### Refinement

**Table:**

Refinement on *F*^2^	Primary atom site location: structure-invariant direct methods
Least-squares matrix: full	Secondary atom site location: difference Fourier map
*R*[*F*^2^ > 2σ(*F*^2^)] = 0.040	Hydrogen site location: inferred from neighbouring sites
*wR*(*F*^2^) = 0.115	H-atom parameters constrained
*S* = 0.94	*w* = 1/[σ^2^(*F*_o_^2^) + (0.0552*P*)^2^] where *P* = (*F*_o_^2^ + 2*F*_c_^2^)/3
1276 reflections	(Δ/σ)_max_ < 0.001
96 parameters	Δρ_max_ = 0.11 e Å^−^^3^
0 restraints	Δρ_min_ = −0.17 e Å^−^^3^

### Special details

**Table:**

Geometry. All e.s.d.'s (except the e.s.d. in the dihedral angle between two l.s. planes) are estimated using the full covariance matrix. The cell e.s.d.'s are taken into account individually in the estimation of e.s.d.'s in distances, angles and torsion angles; correlations between e.s.d.'s in cell parameters are only used when they are defined by crystal symmetry. An approximate (isotropic) treatment of cell e.s.d.'s is used for estimating e.s.d.'s involving l.s. planes.
Refinement. Refinement of *F*^2^ against ALL reflections. The weighted *R*-factor *wR* and goodness of fit *S* are based on *F*^2^, conventional *R*-factors *R* are based on *F*, with *F* set to zero for negative *F*^2^. The threshold expression of *F*^2^ > σ(*F*^2^) is used only for calculating *R*-factors(gt) *etc*. and is not relevant to the choice of reflections for refinement. *R*-factors based on *F*^2^ are statistically about twice as large as those based on *F*, and *R*- factors based on ALL data will be even larger.

### Fractional atomic coordinates and isotropic or equivalent isotropic displacement parameters (Å^2^)

**Table:**

	*x*	*y*	*z*	*U*_iso_*/*U*_eq_	Occ. (<1)
O1	0.78668 (11)	−0.0453 (3)	0.56693 (7)	0.0686 (5)	
O2	0.64734 (11)	−0.0622 (4)	0.49904 (8)	0.0698 (5)	
H2	0.6756	−0.1758	0.4813	0.080*	
C1	0.5000	0.6425 (6)	0.7500	0.0519 (9)	
H1A	0.5479	0.5215	0.7752	0.080*	0.50
H1B	0.4521	0.5215	0.7248	0.080*	0.50
C2	0.54479 (15)	0.8179 (4)	0.70454 (9)	0.0514 (6)	
H2A	0.4973	0.9372	0.6784	0.080*	
H2B	0.5931	0.9392	0.7291	0.080*	
C3	0.58765 (16)	0.6279 (4)	0.66203 (10)	0.0443 (6)	
C4	0.53260 (16)	0.5110 (5)	0.60728 (11)	0.0543 (6)	
H4	0.4695	0.5617	0.5951	0.080*	
C5	0.56894 (16)	0.3211 (5)	0.57026 (10)	0.0522 (6)	
H5	0.5303	0.2454	0.5338	0.080*	
C6	0.66296 (15)	0.2426 (4)	0.58731 (10)	0.0426 (5)	
C7	0.71907 (16)	0.3612 (5)	0.64125 (11)	0.0543 (6)	
H7	0.7824	0.3129	0.6530	0.080*	
C8	0.68157 (17)	0.5525 (5)	0.67812 (10)	0.0552 (6)	
H8	0.7203	0.6309	0.7142	0.080*	
C9	0.70274 (16)	0.0329 (4)	0.54925 (10)	0.0465 (6)	

### Atomic displacement parameters (Å^2^)

**Table:**

	*U*^11^	*U*^22^	*U*^33^	*U*^12^	*U*^13^	*U*^23^
O1	0.0486 (10)	0.0791 (12)	0.0795 (12)	0.0089 (9)	0.0173 (9)	−0.0169 (10)
O2	0.0677 (11)	0.0776 (12)	0.0656 (11)	0.0136 (10)	0.0178 (10)	−0.0211 (10)
C1	0.067 (2)	0.0450 (18)	0.0520 (19)	0.000	0.0308 (18)	0.000
C2	0.0647 (15)	0.0428 (12)	0.0544 (14)	0.0017 (11)	0.0301 (13)	0.0015 (11)
C3	0.0562 (14)	0.0385 (12)	0.0441 (13)	0.0000 (11)	0.0239 (12)	0.0053 (11)
C4	0.0504 (14)	0.0615 (15)	0.0524 (14)	0.0090 (12)	0.0144 (13)	−0.0025 (12)
C5	0.0536 (14)	0.0584 (15)	0.0448 (13)	0.0060 (12)	0.0113 (12)	−0.0049 (11)
C6	0.0476 (13)	0.0419 (12)	0.0419 (13)	0.0000 (11)	0.0178 (11)	0.0033 (10)
C7	0.0469 (13)	0.0603 (15)	0.0574 (14)	0.0035 (12)	0.0148 (12)	−0.0039 (13)
C8	0.0561 (15)	0.0594 (14)	0.0505 (14)	0.0008 (13)	0.0125 (13)	−0.0079 (12)
C9	0.0511 (15)	0.0471 (14)	0.0438 (14)	−0.0032 (12)	0.0158 (13)	0.0003 (11)

### Geometric parameters (Å, °)

**Table:**

O1—C9	1.254 (2)	C3—C4	1.384 (3)
O2—C9	1.278 (2)	C4—C5	1.380 (3)
O2—H2	0.8200	C4—H4	0.9300
C1—C2	1.532 (2)	C5—C6	1.388 (3)
C1—C2^i^	1.532 (2)	C5—H5	0.9300
C1—H1A	0.9700	C6—C7	1.381 (3)
C1—H1B	0.9700	C6—C9	1.482 (3)
C2—C3	1.511 (3)	C7—C8	1.391 (3)
C2—H2A	0.9700	C7—H7	0.9300
C2—H2B	0.9700	C8—H8	0.9300
C3—C8	1.382 (3)		
			
C9—O2—H2	109.5	C5—C4—H4	119.2
C2—C1—C2^i^	114.4 (2)	C3—C4—H4	119.2
C2—C1—H1A	108.7	C4—C5—C6	120.3 (2)
C2^i^—C1—H1A	108.7	C4—C5—H5	119.9
C2—C1—H1B	108.7	C6—C5—H5	119.9
C2^i^—C1—H1B	108.7	C7—C6—C5	118.7 (2)
H1A—C1—H1B	107.6	C7—C6—C9	120.2 (2)
C3—C2—C1	110.64 (17)	C5—C6—C9	121.1 (2)
C3—C2—H2A	109.5	C6—C7—C8	120.5 (2)
C1—C2—H2A	109.5	C6—C7—H7	119.8
C3—C2—H2B	109.5	C8—C7—H7	119.8
C1—C2—H2B	109.5	C3—C8—C7	121.1 (2)
H2A—C2—H2B	108.1	C3—C8—H8	119.4
C8—C3—C4	117.7 (2)	C7—C8—H8	119.4
C8—C3—C2	121.4 (2)	O1—C9—O2	122.9 (2)
C4—C3—C2	120.8 (2)	O1—C9—C6	120.3 (2)
C5—C4—C3	121.7 (2)	O2—C9—C6	116.7 (2)

Symmetry codes: (i) −*x*+1, *y*, −*z*+3/2.

### Hydrogen-bond geometry (Å, °)

**Table:**

*D*—H···*A*	*D*—H	H···*A*	*D*···*A*	*D*—H···*A*
O2—H2···O1^ii^	0.82	1.84	2.642 (2)	167.6

Symmetry codes: (ii) −*x*+3/2, −*y*−1/2, −*z*+1.
